# Supplementary material for: A smooth tubercle bacillus from Ethiopia phylogenetically close to the Mycobacterium tuberculosis complex
Source: Nat Commun. 2023 Nov 18;14:7519. doi: 10.1038/s41467-023-42755-9 (PMC10657438; doi:10.1038/s41467-023-42755-9)
Supplement: Supplementary file 1 — Supplementary Information [file 41467_2023_42755_MOESM1_ESM.docx]

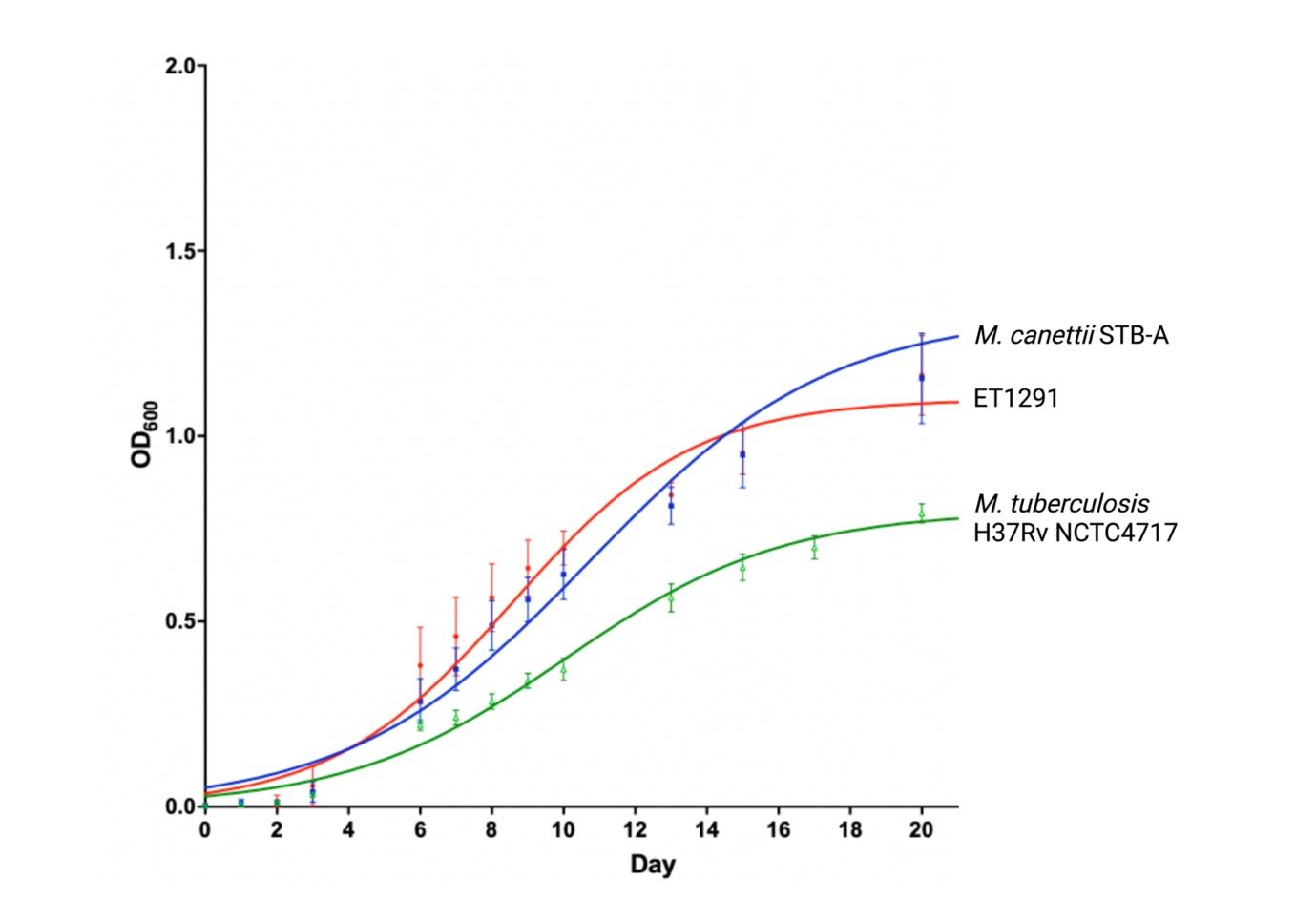


**Supplementary Fig.1.** Growth curves of ET1291, *M. canettii* STB-A and *M. tuberculosis* H37Rv in Middlebrook 7H9 medium supplemented with OADC and 0,5 % glycerol. Measurements of optical densities (OD_600_) over time are shown as mean plus standard deviation from three independent experiments. ET1291 shows a faster growth rate than *M. tuberculosis* H37Rv, similarly to a reference *M. canettii* strain STB-A (CIPT 140010059). The data was analyzed using PRISM 9 software (GraphPad Software, San Diego, CA, USA). Source data are provided as a Source Data file.


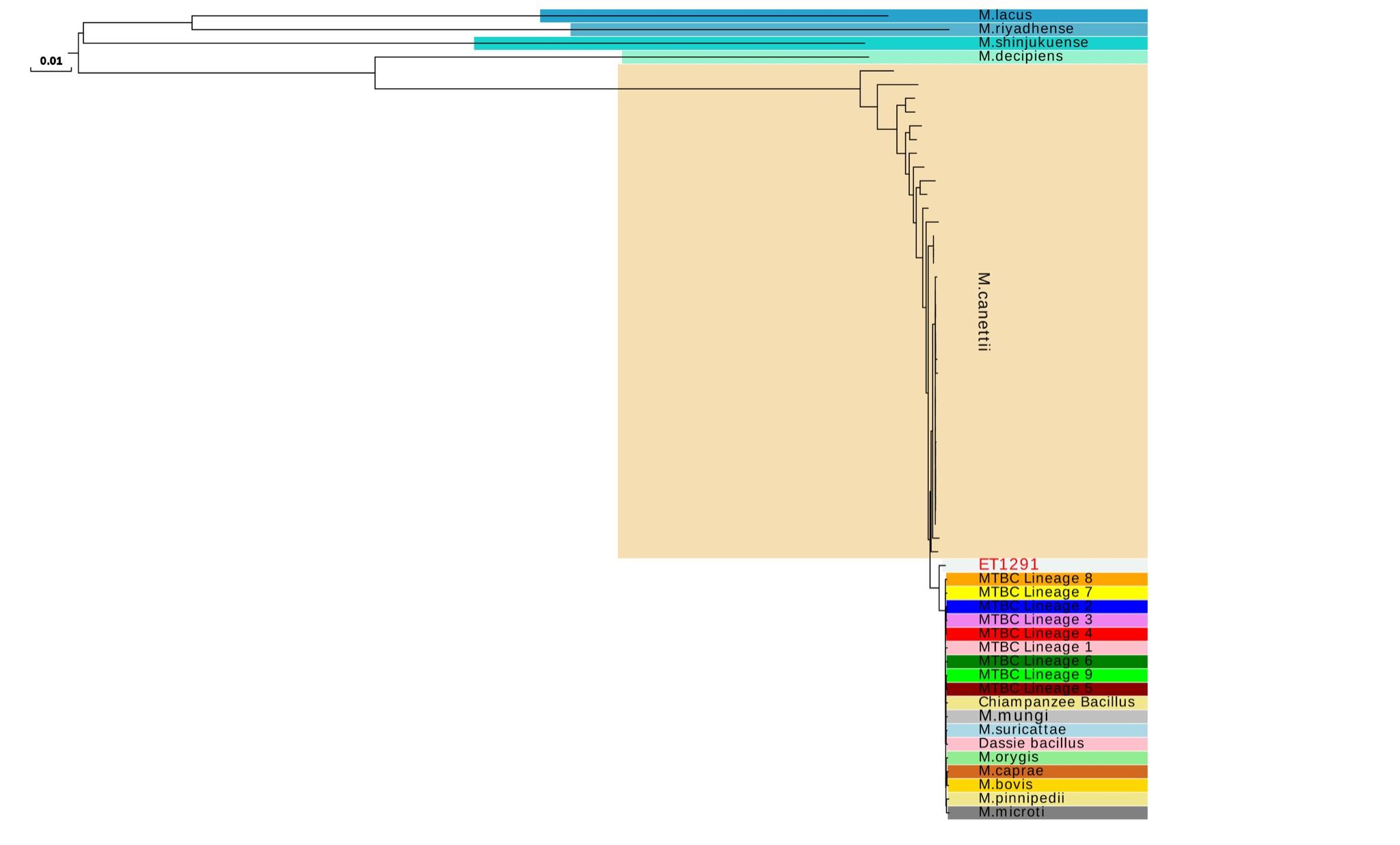


**Supplementary Fig.2.** Core genome-based phylogenetic reconstruction based on 1000 single copy genes of strain representatives of all known human- and animal-adapted lineages of the MTBC, 39 known publicly available or newly sequenced *M. canettii* strains, and phylogenetically closest non-tuberculous mycobacterial species, comprising *M. decipiens, M. shinjukuense, M. lacus and M. riyadhense* (forming a clade defined as the “MTB-associated phylotype”). The intermediate phylogenetic position of the ET1291 branch between the previously known *M. canettii* strains and the MTBC is denoted in red.

**
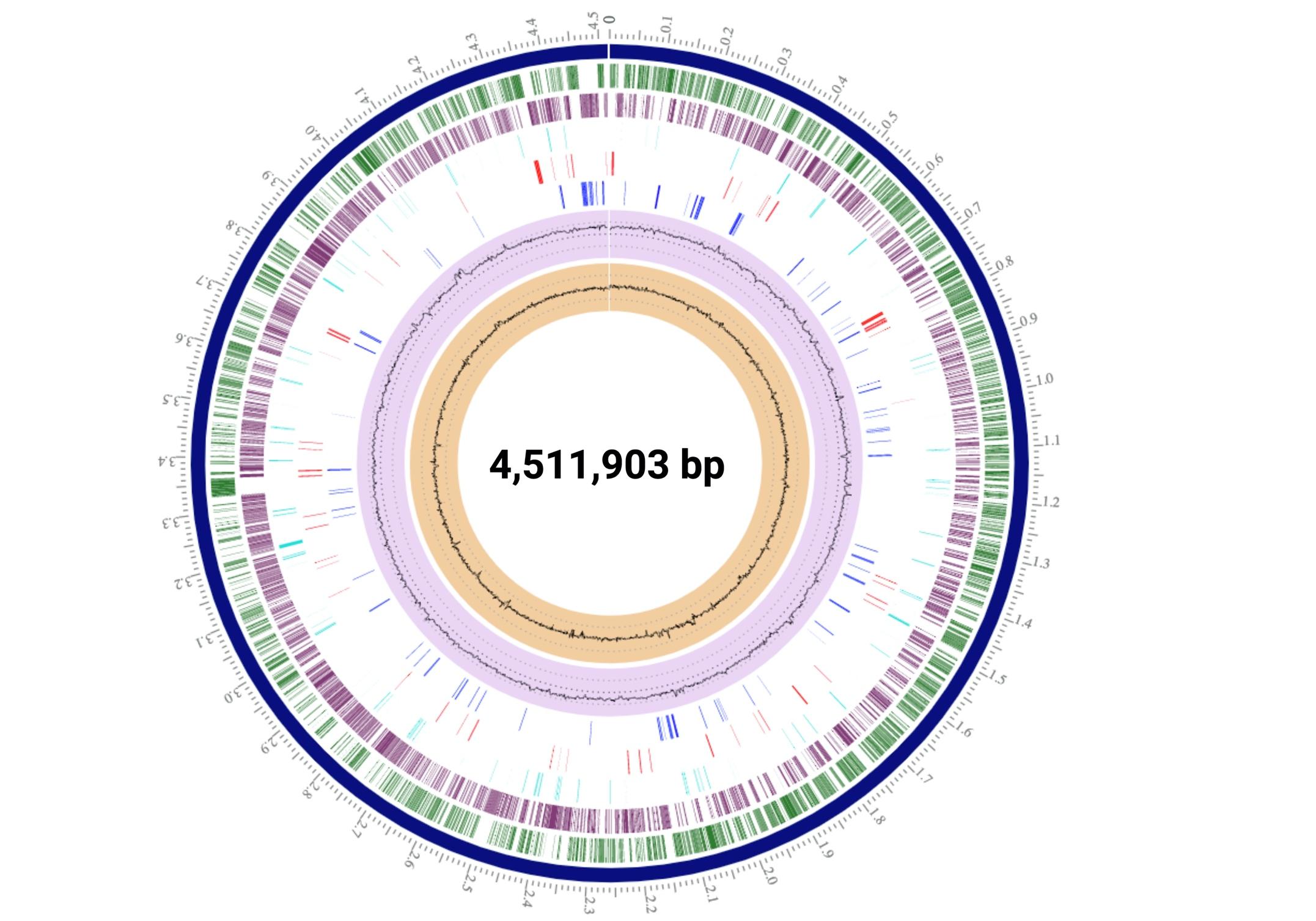
**

**Supplementary Fig.3.** Circular map of the reconstructed complete genome of ET1291. The ET1291 genome has a size of 4,515,631 bp which is is ~85-175 kb larger than the 4.34–4.43 Mb size range of the MTBC genomes. The outer circle shows the scale in Mbp, with 0 representing the origin of replication. The first ring from the exterior denotes the single contig obtained from hybrid assembly of Illumina and Nanopore reads; the second and third rings inwards shows coding sequences (CDS) by strand (clockwise, dark green; anticlockwise, violate); the fourth ring depicts non-CDS features, such as CRISPR spacers/repeats and other repeat regions; the fifth ring shows the positions of known anti-TB drug resistance-associated genes and the sixth ring shows positions of membrane transport protein coding genes (dark blue) based on the classification of Functional and Phylogenetic Classification of Membrane Transport Proteins Database (https://www.tcdb.org/). The two histograms (center) represent the G+C content (pink) and G+C skew (yellow). The figure was generated with the genome viewer feature of the Bacterial And Viral Bioinformatics Resource Center.

**
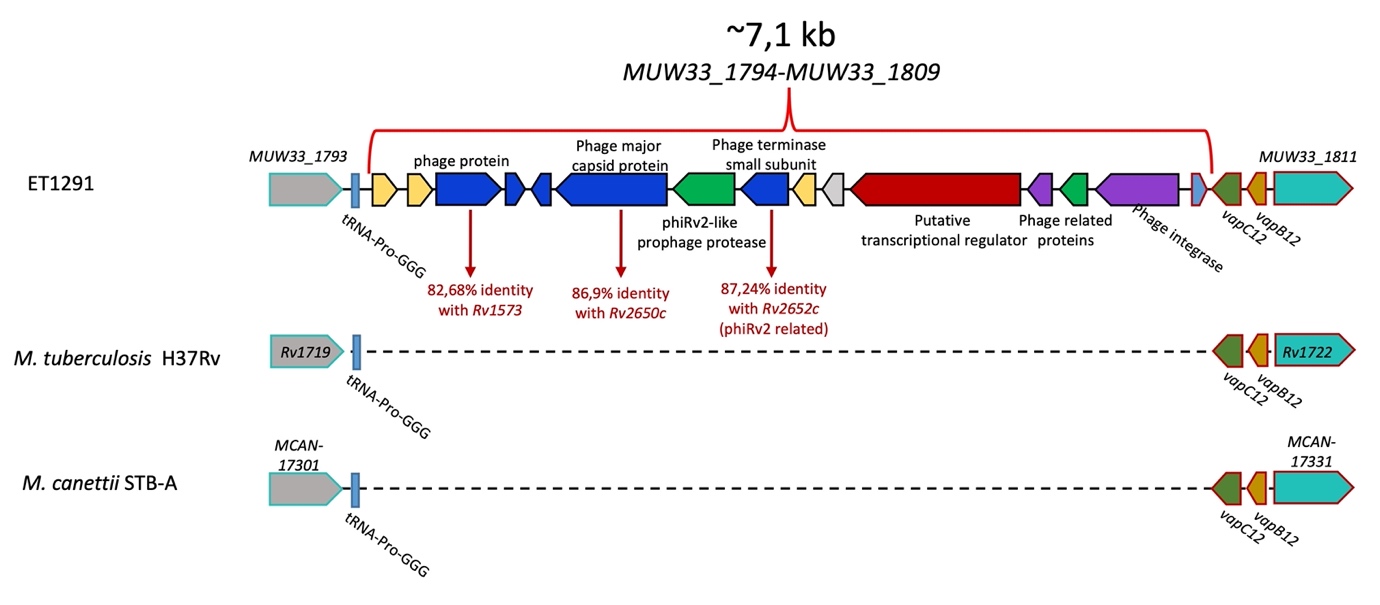
**

**Supplementary Fig.4.** Unique PhiRv-like prophage region in the ET1291 genome, between the orthologues of Rv1719-Rv1720c in H37Rv. Colors define the features of the predicted encoded products: gray, phage protein without database match; CDS in blue, green, yellow, violet and red encode phage proteins homologous (with 71-87% sequence identity) to mycobacteriophage proteins or other proteins of unknown function in MTBC (with specific percentages of identity indicated), *M. canettii* STB-K, *M. shinjukuense*, *M. koreense* and *M. heckeshornense,* respectively. Predicted functions of the proteins are shown below each CDS, except for hypothetical proteins.


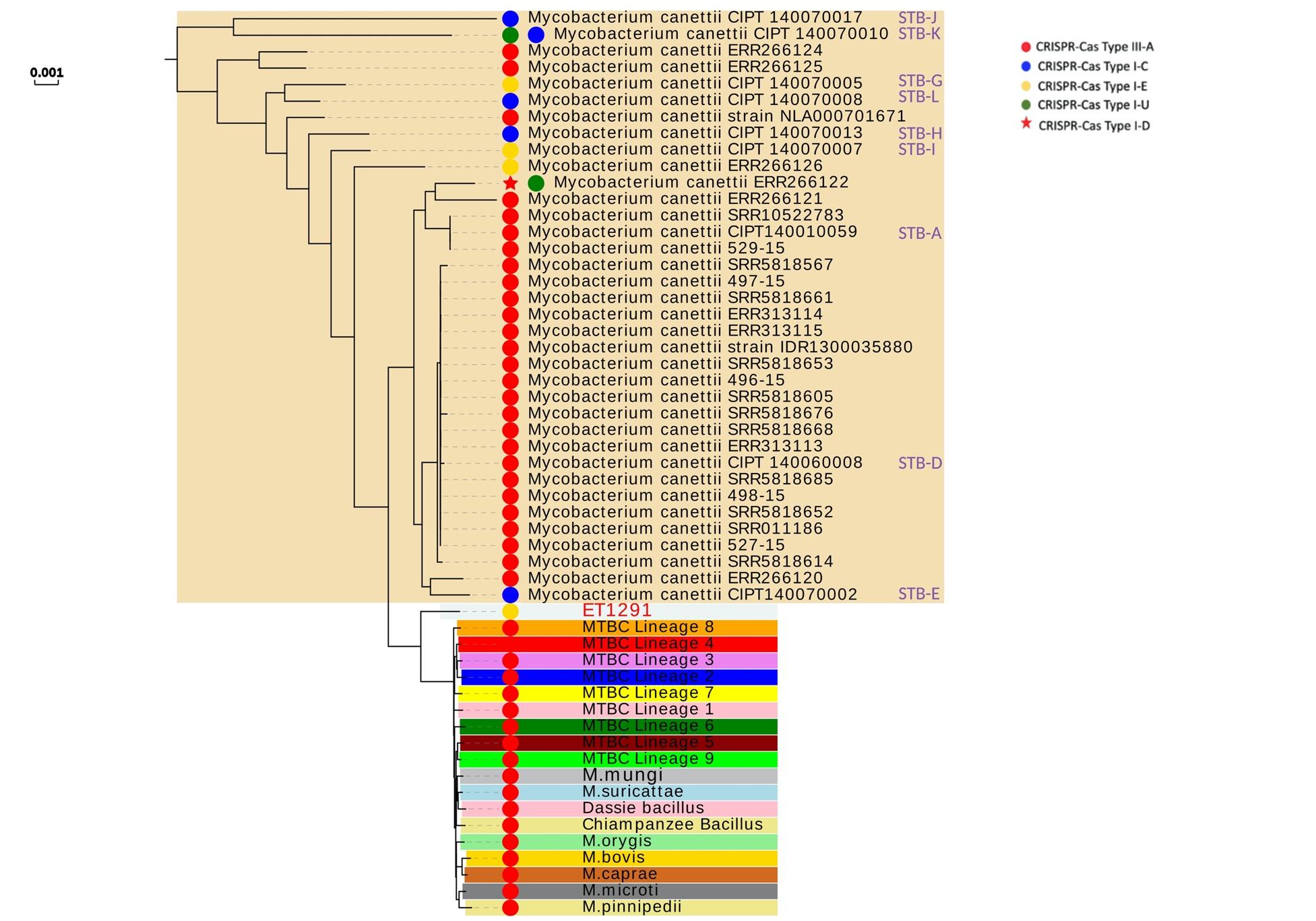


**Supplementary Fig.5.** Distribution of CRISPR-cas systems among MTBC L1-L9, animal-associated lineages of the MTBC, and *M. canettii* strains including ET1291. This distribution is mapped on a core genome-based phylogenetic tree calculated based on 1000 single copy genes. The homoplasic distribution observed in the core genome-based phylogeny indicates multiple independent acquisition events mediated by horizontal gene transfer events, including in the branches leading to ET1291 and/or the MTBC.


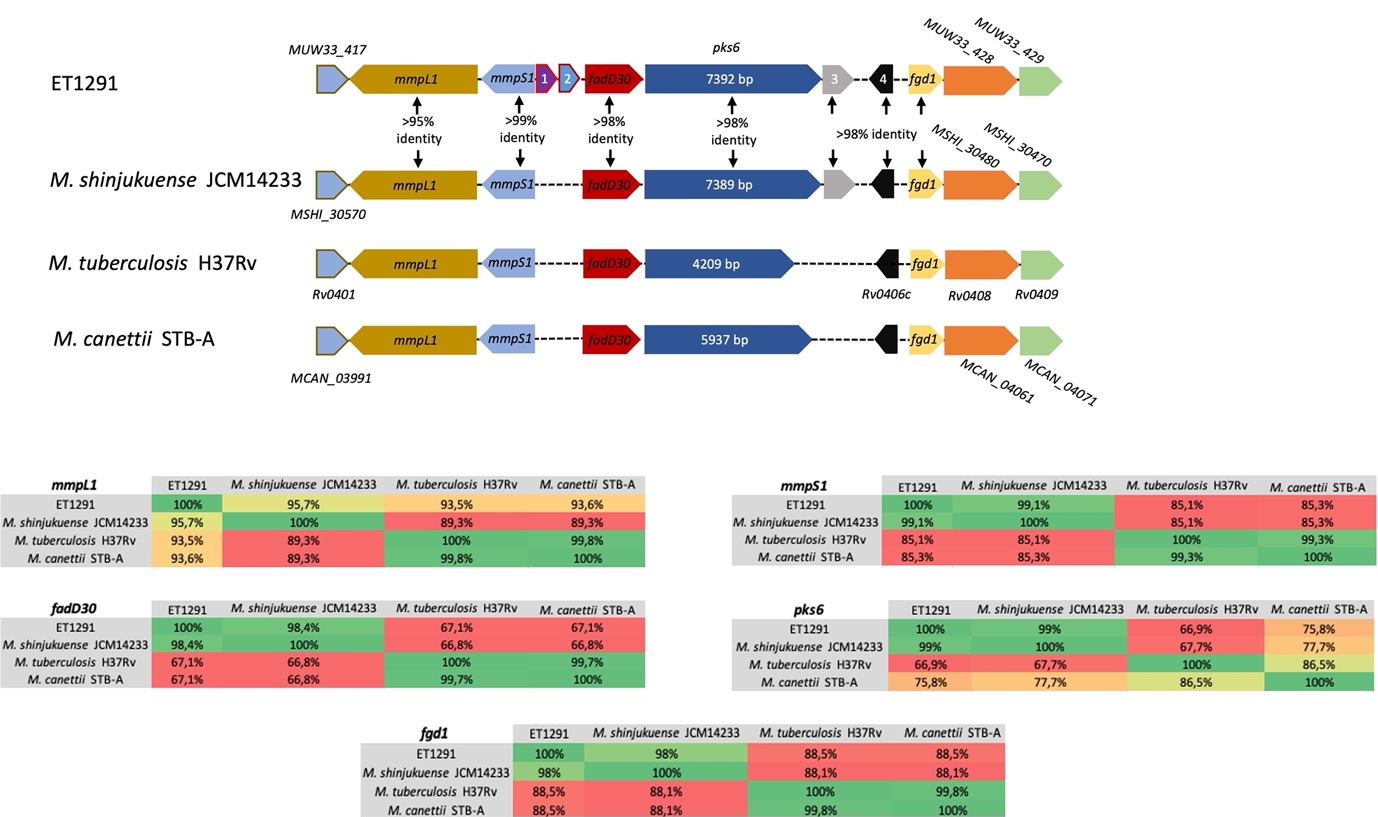


**Supplementary Fig.6.** Top : Schematic overview of the genomic region containing *mmpL1, mmpS1, fadD30, pks6* and *fgd1* genes, showing a higher degree of coding sequence (CDS) identities between ET1291 and *M. shinjukuense,* compared to ET1291 and *M. tuberculosis* H37Rv or *M. canettii* STB-A. Bottom : Cross-comparisons of percent identities of main CDSs between the 4 strains. CDSs numbered 1 & 2 are from a mobile genetic element, and share 98% identity compared to their orthologues in *M. shinjukuense*, containing five copies of this mobile genetic element in other genome regions compared to ET1291. CDSs numbered 3 & 4 code for proteins of unknown function in ET1291 and show >98% identity compared to their orthologues in *M. shinjukuense.* While orthologues of CDS number 4 are also present in *M. canettii* and the MTBC (with lower sequence identities versus ET1291, compared to identities between ET1291 and *M. shinjukuense*), CDS 3 was found only in ET1291 and *M. shinjukuense*.


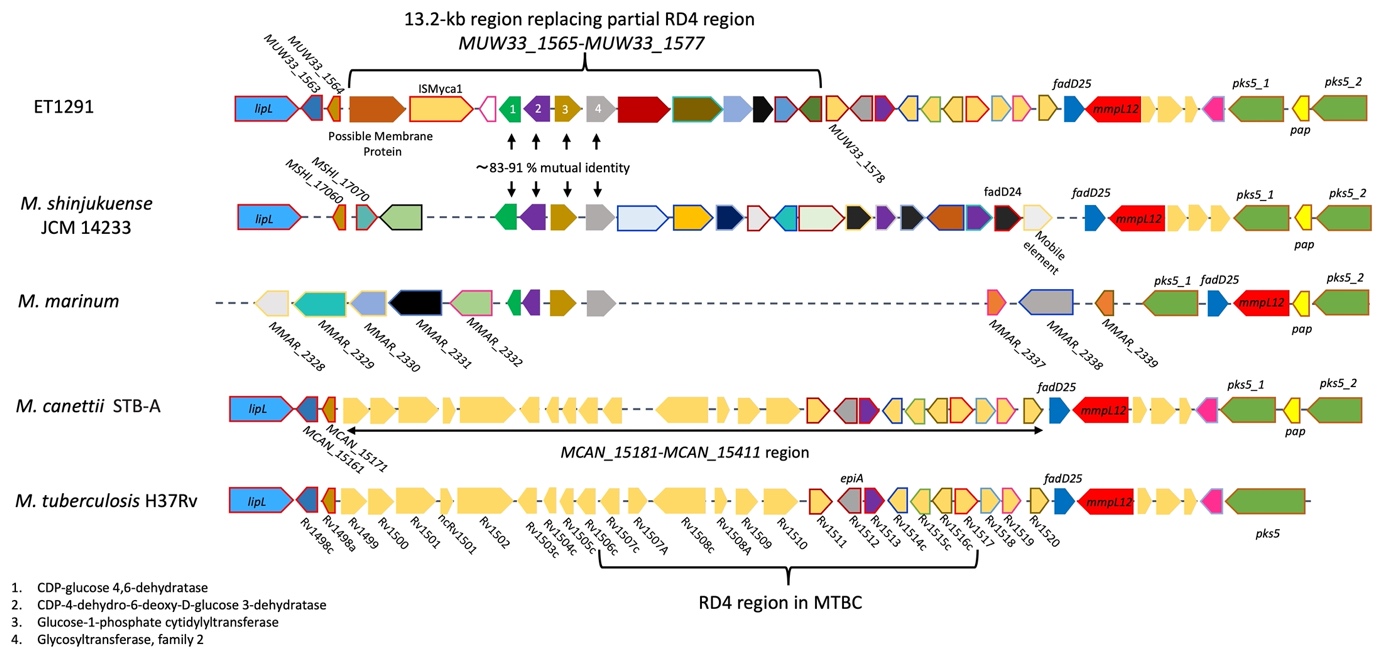


**Supplementary Fig.7.** Schematic overview of an 13.2 kb genomic island in ET1291 unrelated to the segment in the same region in MTBC genomes. This region is found in the ET1291 genome in place of the *Rv1499–1510* region (partial RD4, putatively involved in the biosynthesis of trehalose-containing glycolipids) in *M. tuberculosis* H37Rv. This segment contains 13 genes, of which four (numbered as 1 to 4) with 83-91% and 75-80% coding sequence identities versus their orthologues in *M. shinjukuense* and *M. marinum*, respectively. Colors define the features of the orthologous genes among the strains.

**
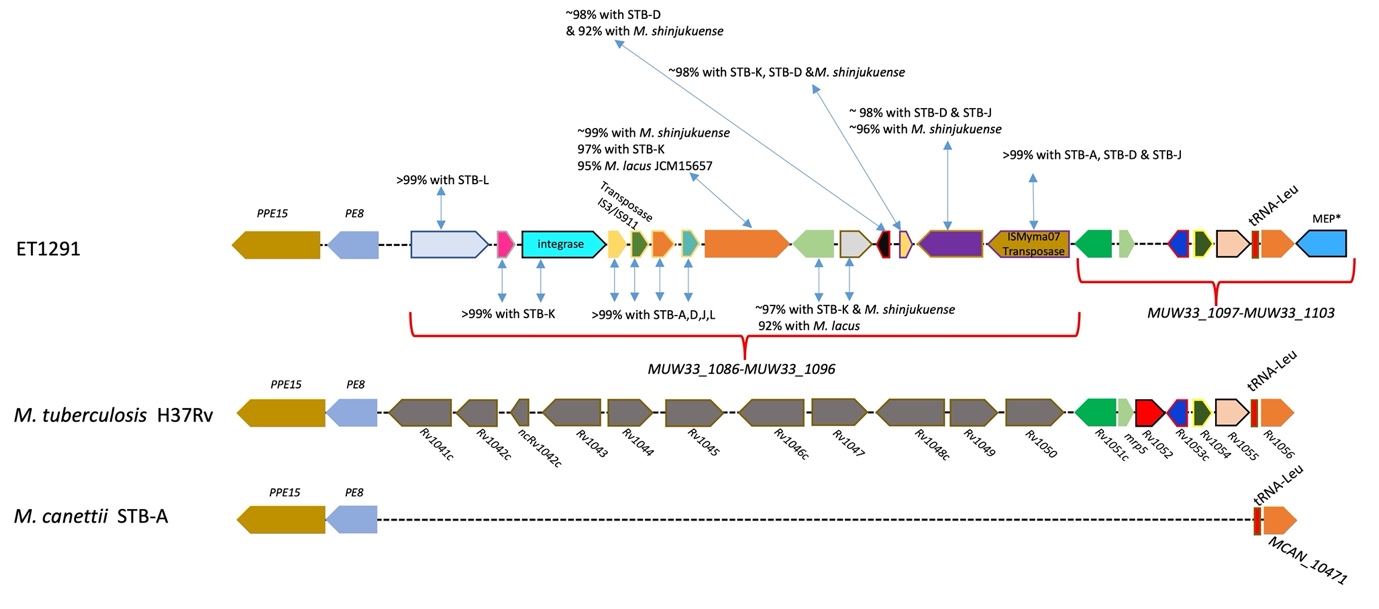
**

**Supplementary Fig.8.** A segment of 15 genes found in the ET1291 genome in place of the *Rv1041c-Rv1050* region in the MTBC genomes. Percentages of coding sequence identities in ET1291 are shown relatively to homologues found in this or other genome regions of some *M. canettii* strains, and *M. shinjukuense* and/or *M. lacus* part of the MTB-associated phylotype. This portion of genome appears particularly variable among *M. canettii* strains (including ET1291) and the MTBC^1^. Indeed, orthologues of *Rv1041c-Rv1050* are present in some *M. canettii* strains such as STB-G, -H and -I, while this region is largely or totally absent in other *M. canettii* strains, such as STB-A, -D, -E, -J, -K and -L. The 13-kb long *Rv1041c-Rv1055* region in the MTBC is a typical example of genomic island, as it is flanked by two 25-bp direct repeats and is inserted in the vicinity of a tRNA sequence (leuX tRNA), even if the GC content of this region is similar to that of the rest of the genome. Also interestingly, *M. tuberculosis* mutants in *Rv1045* and *Rv1046c* were shown to be attenuated in macrophage and SCID mouse infection models, respectively^2,3^. Furthermore, the *Rv1049* gene encodes a transcriptional repressor of the MarR family, and regulators of this family have been implicated in the control of virulence genes in other bacteria^4^. This region fitting characteristics of a pathogenicity island was putatively acquired by the progenitor of the MTBC after divergence from a common ancestor shared with ET1291.

**Supplementary References:**

1. Becq, J. *et al.* Contribution of Horizontally Acquired Genomic Islands to the Evolution ofthe Tubercle Bacilli. *Molecular Biology and Evolution* **24**, 1861–1871 (2007).
2. Rengarajan, J., Bloom, B.R., Rubin, E.J. Genome-wide requirements for Mycobacterium tuberculosis adaptation and survival in macrophages. *Proc Natl Acad Sci U S A* **102**, 8327-8332 (2005).
3. McAdam, R.A. *et al*. Characterization of a Mycobacterium tuberculosis H37Rv transposon library reveals insertions in 351 ORFs and mutants with altered virulence. Microbiology (Reading) **148**, 2975-2986 (2002).
4. Gupta, A. *et al.* MarR Family Transcription Factors from Burkholderia Species: Hidden Clues to Control of Virulence-Associated Genes. *Microbiol Mol Biol Rev* **83**, e00039-18 (2019).
