# Supplementary material for: A smooth tubercle bacillus from Ethiopia phylogenetically close to the Mycobacterium tuberculosis complex
Source: Nat Commun. 2023 Nov 18;14:7519. doi: 10.1038/s41467-023-42755-9 (PMC10657438; doi:10.1038/s41467-023-42755-9)
Supplement: Supplementary file 3 — Description of Additional Supplementary Files [file 41467_2023_42755_MOESM3_ESM.pdf]

Description of Additional Supplementary Files:

Supplementary Data 1: Phylogenetic SNPs found in the ET1291 genome sequence according to analysis using the MTBSeq pipeline

Supplementary Data 2: SNPs found in the fgd1 (Rv0407) and Rv0678 genes in ET1291

Supplementary Data 3: List of the whole genome sequencing data of MTBC, M. canettii and MTB-associated phylotype strains used in this study

Supplementary Data 4: Annotation of the ET1291 genome sequence

Supplementary Data 5: Orthologues in ET1291 of interrupted coding sequences in MTBC genomes

Supplementary Data 6: Average nucleotide identity (ANI) values of ET1291 and other M. canettii strains vs M. tuberculosis H37Rv

Supplementary Data 7: List of the orthologues of eight (shown in red) of the 50 MTBC genes that are present in ET1291 but were not found in the previously identified M. canettii genomes

Supplementary Data 8: Blast analysis of 7/50 MTBC genes present in ET1291 but not found in the previously identified M. canettii genomes, showing top hits with other (myco)bacteria

Supplementary Data 9: Blast analysis of the third prophage region in ET1291 genome
