# Supplementary material for: A smooth tubercle bacillus from Ethiopia phylogenetically close to the Mycobacterium tuberculosis complex
Source: Nat Commun. 2023 Nov 18;14:7519. doi: 10.1038/s41467-023-42755-9 (PMC10657438; doi:10.1038/s41467-023-42755-9)
Supplement: Supplementary file 13 — Reporting Summary [file 41467_2023_42755_MOESM13_ESM.pdf]

Corresponding author(s): Daniela Maria Cirillo, Philip Supply , Arash Ghodousi

Last updated by author(s): 2023/09/22

Reporting Summary

Nature Portfolio wishes to improve the reproducibility of the work that we publish. This form provides structure for consistency and transparency in reporting. For further information on Nature Portfolio policies, see our Editorial Policies and the Editorial Policy Checklist.

Statistics

For all statistical analyses, confirm that the following items are present in the figure legend, table legend, main text, or Methods section.

n/a	Confirmed	
☐	☒	The exact sample size (*n*) for each experimental group/condition, given as a discrete number and unit of measurement
☐	☒	A statement on whether measurements were taken from distinct samples or whether the same sample was measured repeatedly
☒	☐	The statistical test(s) used AND whether they are one- or two-sided<br>*Only common tests should be described solely by name; describe more complex techniques in the Methods section.*
☒	☐	A description of all covariates tested
☒	☐	A description of any assumptions or corrections, such as tests of normality and adjustment for multiple comparisons
☐	☒	A full description of the statistical parameters including central tendency (e.g. means) or other basic estimates (e.g. regression coefficient) AND variation (e.g. standard deviation) or associated estimates of uncertainty (e.g. confidence intervals)
☒	☐	For null hypothesis testing, the test statistic (e.g. *F*, *t*, *r*) with confidence intervals, effect sizes, degrees of freedom and *P* value noted<br>*Give P values as exact values whenever suitable.*
☒	☐	For Bayesian analysis, information on the choice of priors and Markov chain Monte Carlo settings
☒	☐	For hierarchical and complex designs, identification of the appropriate level for tests and full reporting of outcomes
☒	☐	Estimates of effect sizes (e.g. Cohen's *d*, Pearson's *r*), indicating how they were calculated

Our web collection on statistics for biologists contains articles on many of the points above.

Software and code

Policy information about availability of computer code

Data collection	No software was used for data collection
Data analysis	In order to detect drug resistance associated mutations and identify strain (sub)lineages, the sequenced raw reads underwent adapter trimming with Trimmomatic and reads shorter than 20 bp were discarded. To identify and merge any overlapping paired-end reads, the SeqPrep software v1.2 (https://github.com/jstjohn/SeqPrep) was used. The resulting reads were aligned to M. tuberculosis H37Rv ATCC 27294 (NC_000962.3) as reference genomes using the MTBseq pipeline by application of the mem algorithm of the Burrows-Wheeler alignment tool v0.7.17. Duplicated reads were marked using the Picard tool 2.23.4-0 (https://github.com/broadinstitute/picard) and local realignment of reads around InDels was performed using the Genome Analysis Toolkit v3.8. SNPs were called with Samtools mpileup v1.6 using the following thresholds: minimum mapping quality of 20, minimum base quality at a position of 20, minimum read depth at a position of 8X, maximum strand bias for a position of 90%.Genome-wide SNP calling was performed using the CLC Genomics Workbench (version 21.0.5; Qiagen). A custom script was used to extract the positions commonly covered in all genomes, to avoid biases due to variable read coverages across the genome dataset, and to analyze recombination and compare inter-strain SNP distances based on a same, normalized number of sequence positions. PhyML v3.3 was used to build the phylogeny. Bootstrap support values were computed using the PhyML default Shimodaira-Hasegawa-like (SH-like) procedure. This phylogeny was then used as the starting point to build a recombination-corrected phylogeny using ClonalFrameML v1.12. For each branch, the relative effect of recombination versus mutation (r/m) was computed using the formula r/m=(d1-d2)/d2 where d1 and d2 are the lengths of the branches before and after correcting for recombination, respectively. A phylogeny additionally including more distantly related genomes of the MTB-associated phylotype was inferred by using the Codon Tree pipeline of the Bacterial and Viral Bioinformatics Resource Center (BV-BRC, including tools from the previous PATRIC resources), available at https://www.bv-brc.org/app/PhylogeneticTree. The pipeline uses concatenated nucleotide and encoded amino acid sequences from up to 1000 single-copy core genes, identified via detection of cross-genus BV-BRC global Protein Families (PGFams) homology groups , for constructing a RAxML tree. Out of 1160 single-copy core genes identified in all genomes analyzed, 1000 were picked randomly. Corresponding protein and nucleotide coding

sequences were aligned using MUSCLE and the Codon_align function of BioPython, respectively.
The concatenated alignment of all protein and nucleotide sequences written in a PHYLIP file was partitioned for describing the alignment in terms of protein sequences and first, second and third codon positions of nucleotide sequences. The resulting file was used for constructing a maximum-likelihood tree using RAxML-NG v1.0.2 with '–model GTR+G+ASC_LEWIS. We used the general time reversible model of nucleotide substitution under the gamma model of rate heterogeneity and performed 1000 alternative runs on distinct starting trees. Support values were generated using 100 rounds of the "Rapid" bootstrapping and the best-scoring maximum-likelihood topology was "midpoint rooted" using FigTree v1.4.4 (https://github.com/cdeanj/figtree). The topology was annotated and colored using the Evolview v3 online tool.In order to reconstruct a high-quality assembled genome based on the Illumina short reads and ONT long reads, a hybrid genome assembly was constructed as follows. Nanopore long reads were corrected using the Ratatosk de novo error correction tool v0.7.6.3 using Illumina paired-end short reads. Corrected long reads were assembled using Flye de novo assembler v2.9.1 (https://github.com/fenderglass/Flye). Initial genome annotation was done using the BV-BRC RASTtk-enabled genome annotation service. A circular map of the chromosome was visualized with BRIG.In silico spoligotyping was performed with Illumina sequence reads using the kvarQ software v0.12.2 . To detect clustered regularly interspaced short palindromic repeats (CRISPR) -Cas loci in assembled genomes, the CRISPRCasFinder pipeline (https://github.com/dcouvin/CRISPRCasFinder) was used with default parameters. CRISPR-Cas type and subtype were assigned by using CRISPRmap.

For manuscripts utilizing custom algorithms or software that are central to the research but not yet described in published literature, software must be made available to editors and reviewers. We strongly encourage code deposition in a community repository (e.g. GitHub). See the Nature Portfolio guidelines for submitting code & software for further information.

Data

Policy information about availability of data

All manuscripts must include a data availability statement. This statement should provide the following information, where applicable:

- Accession codes, unique identifiers, or web links for publicly available datasets
- A description of any restrictions on data availability
- For clinical datasets or third party data, please ensure that the statement adheres to our policy

The corresponding sequence reads, as well as the Nanopore reads, the associated sequence assembly and annotation (see below), were submitted to the NCBI Sequence Read Archive with Project number PRJNA823537.

Research involving human participants, their data, or biological material

Policy information about studies with human participants or human data. See also policy information about sex, gender (identity/presentation), and sexual orientation and race, ethnicity and racism.

Reporting on sex and gender	N/A, except that the male gender of the TB patient from whom the ET1291 strain was isolated is indicated in the text.
Reporting on race, ethnicity, or other socially relevant groupings	N/A
Population characteristics	The strain was identified within the national anti-TB drug resistance survey 2017-2019, designed as a cross-sectional health facility-based study as per WHO recommendations, enrolling for 16 months newly registered and previously treated bacteriologically confirmed pulmonary TB cases in all age groups diagnosed at the study sites.
Recruitment	The strain was identified within the national anti-TB drug resistance survey 2017-2019, designed as a cross-sectional health facility-based study as per WHO recommendations, enrolling for 16 months newly registered and previously treated bacteriologically confirmed pulmonary TB cases.
Ethics oversight	The ET1291 strain was isolated from a patient with TB in the framework of a national drug resistance survey in Ethiopia according to WHO recommendations.

Note that full information on the approval of the study protocol must also be provided in the manuscript.

Field-specific reporting

Please select the one below that is the best fit for your research. If you are not sure, read the appropriate sections before making your selection.

[x] Life sciences [ ] Behavioural & social sciences [ ] Ecological, evolutionary & environmental sciences

For a reference copy of the document with all sections, see nature.com/documents/nr-reporting-summary-flat.pdf

Life sciences study design

All studies must disclose on these points even when the disclosure is negative.

Sample size	To determine the phylogenetic position of ET1291, a maximum likelihood phylogeny was inferred from a whole genome alignment of 80 MTBC genomes, as well as 39 M. canettii genomes. The set of MTBC genomes was selected to include representatives of all known human- and animal-adapted (sub)lineages. The set of M. canettii genomes includes all publicly available genome sequences as well as one newly sequenced genome of this taxon. As an external group to both the MTBC and M. canettii, we selected a set comprising one publicly available genome for each of the following non-tuberculous mycobacterial species, known to be the phylogenetically closest outgroup to the tuberculosis bacilli: M. decipiens, M. shinjukuense, M. lacus and M. riyadhense (forming a clade defined as the "MTB-associated phylotype").

Data exclusions	No Data exclusions
Replication	Bootstrapping procedures were applied to verify the robustness of the phylogenetic reconstruction. For the measurements of mycobacterial growth, the experiments were done in three independent replicates.
Randomization	Bootstrapping procedures were applied to verify the robustness of the phylogenetic reconstruction
Blinding	Blinding was not applicable as the study was not of a case/control type or similar.

Reporting for specific materials, systems and methods

We require information from authors about some types of materials, experimental systems and methods used in many studies. Here, indicate whether each material, system or method listed is relevant to your study. If you are not sure if a list item applies to your research, read the appropriate section before selecting a response.

Materials & experimental systems

n/a	Involved in the study
☒	☐ Antibodies
☒	☐ Eukaryotic cell lines
☒	☐ Palaeontology and archaeology
☒	☐ Animals and other organisms
☒	☐ Clinical data
☒	☐ Dual use research of concern
☒	☐ Plants

Methods

n/a	Involved in the study
☒	☐ ChIP-seq
☒	☐ Flow cytometry
☒	☐ MRI-based neuroimaging
